# Coronary microvascular dysfunction mediates in-hospital adverse outcomes in ST-segment elevation myocardial infarction patients without standard modifiable cardiovascular risk factors

**DOI:** 10.3389/fcvm.2026.1808392

**Published:** 2026-04-17

**Authors:** Guoli Yao, Mengru Guo, Na Song, Jie Liu, Wanling Wu, Defeng Pan, Yuanyuan Luo

**Affiliations:** Department of Cardiology, The Affiliated Hospital of Xuzhou Medical University, Xuzhou, China

**Keywords:** coronary angiography-derived index of microcirculatory resistance, coronary microvascular dysfunction, mediation analysis, standard modifiable cardiovascular disease risk factors, ST-segment elevation myocardial infarction

## Abstract

**Background:**

Recent studies have demonstrated that patients with ST-segment elevation myocardial infarction (STEMI) without standard modifiable cardiovascular risk factors (SMuRF-less) experience worse in-hospital outcomes than those with standard modifiable cardiovascular risk factors (SMuRFs); however, the role of a critical pathological mechanism coronary microvascular dysfunction (CMVD) in this context remains unclear.

**Objectives:**

To investigate whether CMVD mediates the association between SMuRF-less status and in-hospital major adverse cardiovascular events (MACE).

**Methods:**

A total of 1,027 STEMI patients undergoing percutaneous coronary intervention (PCI) were enrolled and classified into a SMuRFs group (≥1 SMuRF, *n* = 806) and a SMuRF-less group (no SMuRFs, *n* = 221). In-hospital MACE and post-procedural coronary angiography–derived index of microcirculatory resistance (caIMR) were compared between groups. Mediation analysis was performed to assess the mediating effect of post-procedural caIMR on the association between SMuRF-less status and in-hospital outcomes.

**Results:**

The incidence of in-hospital MACE was significantly higher in the SMuRF-less group than in the SMuRFs group (15.3% vs. 7.0%, *P* < 0.001). After adjustment for potential confounders, mediation analysis demonstrated that post-procedural caIMR partially mediated the association between SMuRF-less status and increased in-hospital MACE risk (*β* = 0.284, 95% bootstrap CI: 0.101–0.498).

**Conclusion:**

Elevated post-procedural caIMR, as a quantitative surrogate of CMVD, independently predicted in-hospital MACE and partially mediated the association between SMuRF-less status and adverse in-hospital outcomes in STEMI patients.

## 1 Introduction

ST-segment elevation myocardial infarction (STEMI) represents the most severe form of acute myocardial infarction (AMI) and remains a leading cause of mortality among patients with coronary artery disease (CAD) (1). Traditionally, standard modifiable cardiovascular risk factors (SMuRFs), including cigarette smoking, hypertension, diabetes mellitus, and dyslipidemia, have been regarded as the principal drivers of STEMI occurrence and adverse clinical outcomes (2). However, accumulating evidence in recent years has challenged this paradigm, demonstrating that a subset of STEMI patients without these conventional risk factors—referred to as SMuRF-less STEMI—do not exhibit a more favorable prognosis. On the contrary, these patients consistently show higher in-hospital and long-term mortality, as well as increased rates of major adverse cardiovascular events (MACE), compared with their counterparts with SMuRFs (3, 4). This unexpected “prognostic paradox” has been repeatedly observed across multiple countries and large-scale registry studies, yet its underlying mechanisms remain incompletely understood.

Successful restoration of epicardial coronary artery patency does not necessarily translate into effective myocardial reperfusion. Substantial evidence indicates that even in the presence of optimal angiographic results and Thrombolysis in Myocardial Infarction (TIMI) grade 3 flow, a considerable proportion of STEMI patients develop coronary microvascular dysfunction (CMVD), which is closely associated with larger infarct size, impaired left ventricular function, and adverse clinical outcomes (5, 6). In recent years, angiography-based indices of microcirculatory function—particularly the coronary angiography–derived index of microcirculatory resistance (caIMR)—have emerged as practical tools for post-procedural assessment of microvascular function. Owing to the absence of pressure-wire requirements, procedural simplicity, and good agreement with invasively measured IMR, caIMR has gained increasing acceptance in clinical practice (7). Previous studies have consistently demonstrated that elevated post-procedural caIMR is significantly associated with an increased risk of MACE in STEMI patients (8).

Although prior investigations have described the unfavorable prognosis observed in SMuRF-less STEMI populations (9, 10), whether elevated post-procedural caIMR plays a mediating role in the association between SMuRF-less status and adverse clinical outcomes has not been systematically quantified. Therefore, the present study aimed to investigate the relationship between SMuRF-less status and in-hospital adverse outcomes in STEMI patients and to further elucidate, through mediation analysis, the extent to which elevated post-procedural caIMR mediates this association. By focusing on the coronary microcirculation, this study seeks to provide mechanistic insights into the prognostic paradox of SMuRF-less STEMI and to inform refined strategies for risk stratification and clinical management.

## 2 Methods

### 2.1 Study population

This was a single-center, retrospective observational study. The study population was derived from consecutive patients with STEMI who underwent percutaneous coronary intervention (PCI) at the Affiliated Hospital of Xuzhou Medical University between August 1, 2019, and July 31, 2024. A total of 1,959 STEMI patients were initially screened.Inclusion criteria were as follows: (1) age ≥18 years; (2) diagnosis of STEMI according to current clinical guidelines (11); (3) post-procedural coronary angiographic image quality sufficient for calculation of the caIMR; and (4) complete baseline clinical data and in-hospital outcome information. Exclusion criteria included any of the following: (1) a documented history of coronary artery disease; (2) inability to calculate caIMR due to poor angiographic image quality or incomplete data; (3) other conditions deemed unsuitable for study inclusion, including but not limited to cardiogenic shock at presentation, severe valvular heart disease, severe heart failure unrelated to the index myocardial infarction, severe hepatic or renal dysfunction, active infection, inflammatory disease, or malignancy; and (4) missing key clinical variables or in-hospital outcome data.After applying the inclusion and exclusion criteria, a total of 1,027 STEMI patients were included in the final analysis.

### 2.2 Definition of SMuRFs and patient grouping

**Figure 1:**
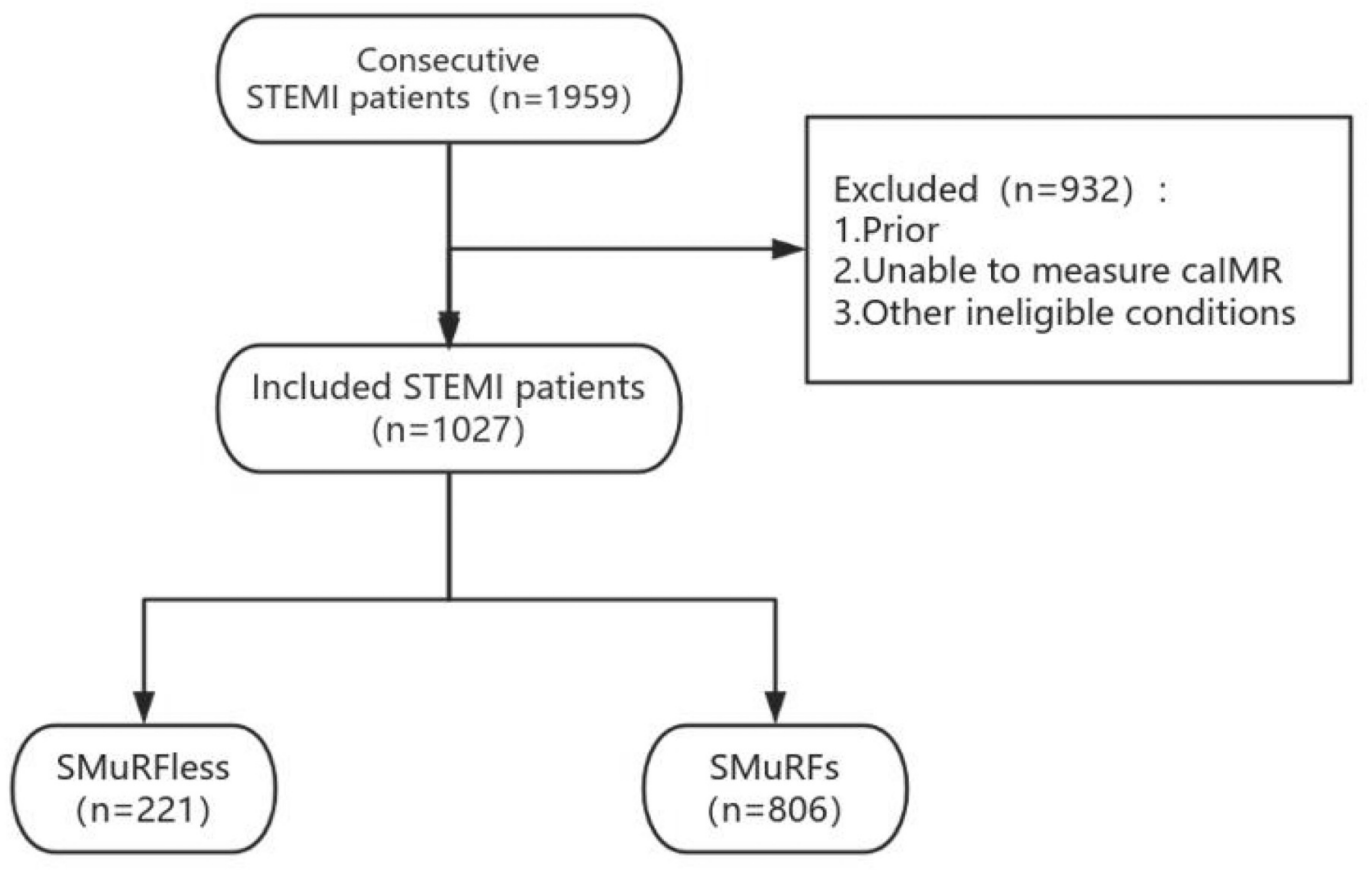
Flowchart of patient selection. STEMI,ST-segment elevation myocardial infarction; caIMR, coronary angiography-derived index of microcirculatory resistance; SMuRFs, standard modifiable cardiovascular risk factors; SMuRF-less, without standard modifiable cardiovascular risk factors.

SMuRFs were defined as a documented history of hypertension, hyperlipidemia, or diabetes mellitus, or current cigarette smoking (12). A documented medical history was defined as patient-reported prior diagnosis or ongoing treatment with relevant medications. Current smoking was defined as regular smoking within the preceding 12 months. Based on the presence or absence of SMuRFs, patients were classified into the SMuRFs group and the SMuRF-less group. The study flowchart is shown in Figure 1. The study protocol was approved by the Institutional Ethics Committee of the Affiliated Hospital of Xuzhou Medical University (approval number: XYFY-2025-KL562-01).

### 2.3 caIMR

**Figure 2:**
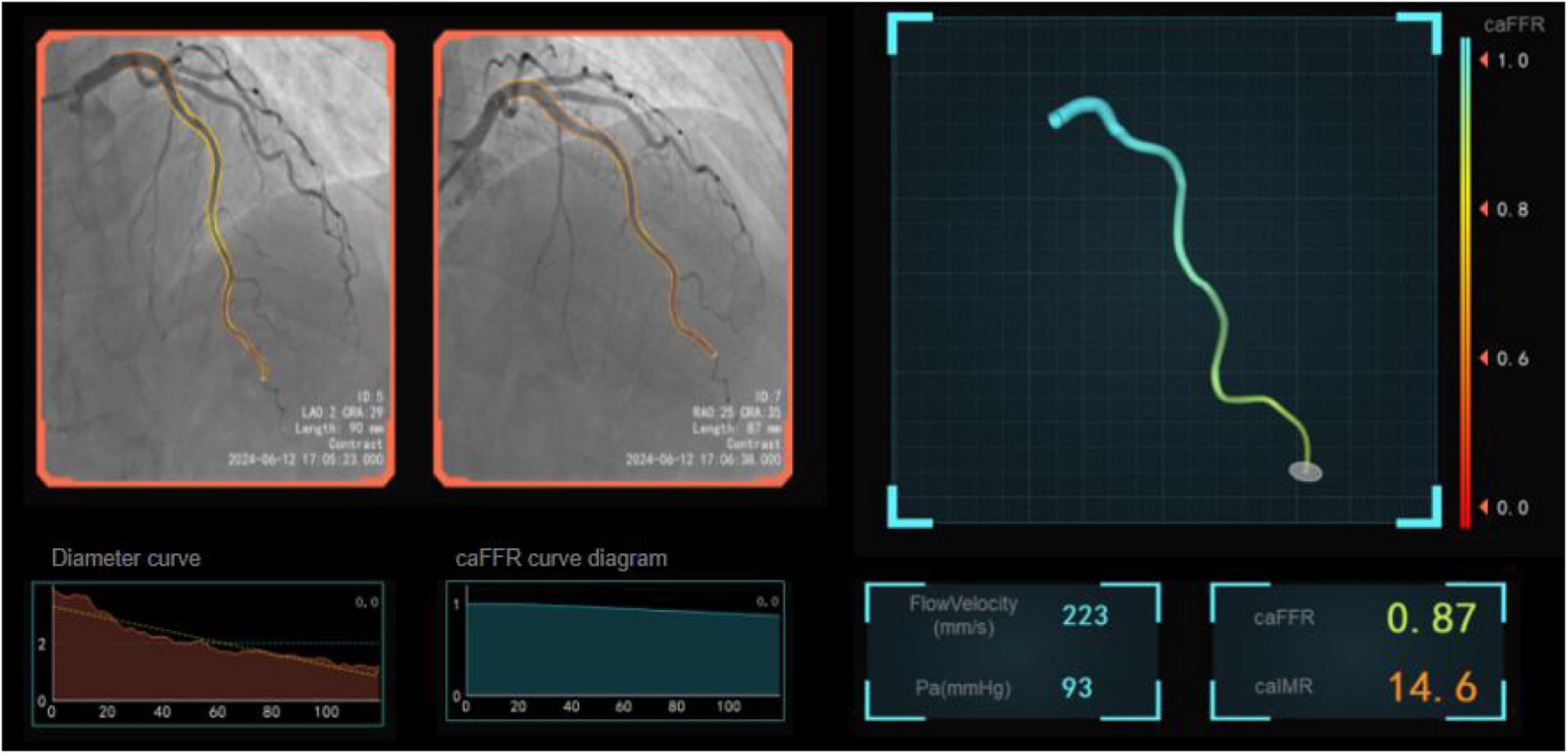
Representative cases of STEMI with post-procedural caIMR measurement. caIMR,coronary angiography–derived index of microcirculatory resistance; caFFR, coronary angiography–derived fractional flow reserve.

The coronary angiography–derived index of microcirculatory resistance (caIMR) of the target vessel was analyzed using the Flash-Angio system (Rainmed Ltd, Suzhou, China). All caIMR measurements were independently performed by two experienced physicians who were blinded to clinical data.The theoretical basis and methodology of caIMR measurement have been described in detail previously (13). The calculation formula is as follows:

$$caIMR=(Pd{)}_{hyp}\frac{L}{K\times V_{diastole}}$$

where (Pd)_hyp_ represents the mean distal coronary pressure during maximal hyperemia (mmHg); L is a constant representing the simulated path length from the coronary ostium to the distal vessel (L = 75); V_diastole_ denotes the mean distal coronary flow velocity during diastole (mm/s); and V_hyp_, the mean distal flow velocity during maximal hyperemia, is calculated as V_hyp_ = K × V_diastole_ (K = 2.1). In patients with severe epicardial coronary stenosis, caIMR was adjusted using Yong's formula (14), which accounts for the potential influence of collateral flow–induced wedge pressure under conditions of significant epicardial stenosis (15). In the present study, caIMR was primarily measured at the culprit lesion site. All patients undergoing caIMR assessment had an identifiable epicardial culprit vessel and achieved successful revascularization following PCI. Based on previous studies (16, 17), CMVD was defined as a post-procedural caIMR > 40 in the present study. An example of caIMR measurement is shown in Figure 2.

### 2.4 Study design

**Figure 3:**
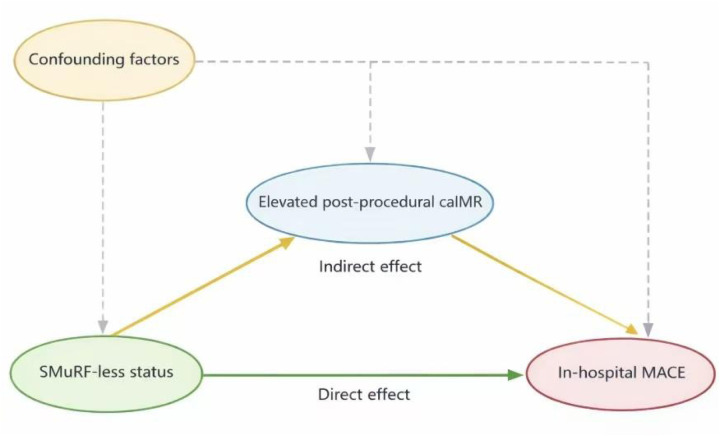
Conceptual model of mediation analysis. Elevated post-procedural caIMR (mediators) and In-hospital MACE (outcome) in the presence of measured confounders. SMuRF-less, without standard modifiable cardiovascular risk factors; caIMR,coronary angiography–derived index of microcirculatory resistance; MACE, major adverse cardiovascular events.

This was a single-center, retrospective observational study that consecutively enrolled patients admitted with STEMI who successfully underwent PCI. Patients were categorized into the SMuRF-less group or the SMuRFs group according to the presence of standard modifiable cardiovascular risk factors. Coronary microvascular function was assessed immediately after PCI using caIMR.The primary clinical endpoint was the occurrence of in-hospital MACE, defined as a composite of all-cause death, heart failure, cardiogenic shock, ischemia-driven revascularization, cardiac arrest, and malignant arrhythmias during hospitalization. Clinical outcomes were defined in accordance with the Academic Research Consortium criteria (18). After adjustment for potential confounders, mediation analysis using the bootstrap method was performed to evaluate the mediating effect of post-procedural caIMR on the association between SMuRF status and in-hospital adverse outcomes. The mediation effect analysis is illustrated in Figure 3.

### 2.5 Statistical analysis

Statistical analyses were performed using R software (version 4.4.1) and SPSS Statistics (version 27.0). Continuous variables are presented as mean ± standard deviation or median (interquartile range), as appropriate. Normality was assessed using the Kolmogorov–Smirnov test and Q–Q plots. Categorical variables are expressed as counts (percentages). Between-group comparisons of continuous variables were conducted using the independent-samples *t*-test or the Mann–Whitney *U*-test, whereas categorical variables were compared using the chi-square test or Fisher's exact test.Mediation analysis was conducted using SPSS 27.0 with the PROCESS macro (version 4.0) (19). In-hospital MACE was specified as the dependent variable, SMuRF status as the independent variable, and post-procedural caIMR as the mediator, with adjustment for age, sex, and other covariates. Bias-corrected percentile bootstrap resampling (5,000 iterations) was applied to estimate 95% confidence intervals for the mediation effect. A mediation effect was considered statistically significant when the 95% bootstrap confidence interval did not include zero.

## 3 Results

### 3.1 Population characteristics

A total of 1,027 STEMI patients were included in the present study, comprising 221 SMuRF-less patients (21.5%) and 806 patients with SMuRFs (78.4%). Compared with the SMuRFs group, the SMuRF-less group had a significantly higher proportion of female patients (28.0% vs. 19.8%, *P* = 0.009) and was significantly older (65.50 ± 11.87 years vs. 59.59 ± 12.58 years, *P* < 0.001).With respect to anthropometric measurements and vital signs, the SMuRF-less group had a significantly lower BMI than the SMuRFs group (23.87 ± 3.72 kg/m^2^ vs. 25.25 ± 3.32 kg/m^2^, *P* < 0.001). Heart rate did not differ significantly between groups (*P* = 0.294); both systolic blood pressure (SBP) and diastolic blood pressure (DBP) were significantly lower in the SMuRF-less group than in the SMuRFs group (both *P* < 0.01). There was no significant difference in symptom-to-door time (STD time) between groups (*P* = 0.235), whereas the SMuRF-less group had a slightly shorter length of stay (LOS) (7.57 ± 4.11 days vs. 8.16 ± 3.38 days, *P* = 0.031). Detailed baseline characteristics are summarized in Table 1.

**Table 1:** Baseline characteristics.

Variables	SMuRF-less	SMuRFs	Total	*P*
	(221, 21.5%)	(806, 78.4%)	(1,027, 100%)	
Female	62 (28.0%)	160 (19.8%)	222 (21.6%)	0.009
Age (year)	65.50 ± 11.87	59.59 ± 12.58	60.86 ± 12.66	<0.001
SMuRF				
Hypertension	0 (0%)	412 (51.1%)	412 (40.1%)	<0.001
Smoking	0 (0%)	408 (50.6%)	408 (39.7%)	<0.001
hyperlipidemia	0 (0%)	365 (45.2%)	365 (35.5%)	<0.001
Diabetes mellitus	0 (0%)	188 (23.3%)	188 (18.3%)	<0.001
BMI (kg/m^2^)	23.87 ± 3.72	25.25 ± 3.32	24.95 ± 3.45	<0.001
HR (times/min)	80.20 ± 14.67	79.09 ± 13.67	79.33 ± 13.89	0.294
SBP (mmHg)	121.22 ± 18.47	124.95 ± 19.46	124.14 ± 19.30	0.008
DBP (mmHg)	74.59 ± 11.86	77.20 ± 13.17	76.63 ± 12.93	0.008
STD time (hour)	5 [3, 15]	7 [4, 23]	6 [4, 20]	0.235
LOS (day)	7.57 ± 4.11	8.16 ± 3.38	8.03 ± 3.55	0.031

SMuRFs, standard modifiable cardiovascular risk factors; SMuRF-less, without standard modifiable cardiovascular risk factors; BMI, body mass index; HR, heart rate; SBP, systolic blood pressure; DBP, diastolic blood pressure; STD time, symptom-to-door time; LOS, length of stay.

Laboratory findings showed no significant differences between groups in white blood cell (WBC) count, inflammatory markers including C-reactive protein (CRP), myocardial injury biomarkers including N-terminal pro–B-type natriuretic peptide (NT-pro-BNP), high-sensitivity cardiac troponin T (hs-TnT), creatine kinase–myocardial band (CK-MB), lactate dehydrogenase (LDH), or indices of hepatic and renal function (all *P* > 0.05), except for uric acid (UA), which was lower in the SMuRF-less group (292.70 ± 100.54 μmol/L vs. 311.17 ± 90.64 μmol/L, *P* = 0.012). As expected, levels of fasting blood glucose (FBG), glycated hemoglobin A1c (HbA1c), total cholesterol (TC), triglycerides (TG), and low-density lipoprotein cholesterol (LDL-C) were significantly lower in the SMuRF-less group than in the SMuRFs group (all *P* < 0.05). Moreover, no significant difference in left ventricular ejection fraction (LVEF) assessed by echocardiography after admission was observed between groups. These data are presented in Supplementary Table S1.

Coronary angiographic findings and interventional characteristics are shown in Supplementary Table S2. Except for a lower proportion of left circumflex coronary artery (LCX) as the culprit vessel in the SMuRF-less group compared with the SMuRFs group (7.6% vs. 14.6%, *P* = 0.007), there were no significant differences between groups in culprit vessel distribution, involvement of the left main artery (LM), severity of culprit vessel stenosis, or Gensini score, or prevalence of multivessel disease (all *P* > 0.05). The distribution of culprit vessel stenosis severity was also similar between the two groups. The SMuRF-less group exhibited a higher proportion of pre-procedural Thrombolysis in Myocardial Infarction (TIMI) flow grade 0 and a lower proportion of pre-procedural TIMI flow grade 3. Procedural duration did not differ significantly between the two groups (all *P* > 0.05).

With respect to pharmacological therapy during hospitalization, the use of statins, low-molecular-weight heparin, angiotensin-converting enzyme inhibitors (ACEIs)/angiotensin II receptor blockers (ARBs)/angiotensin receptor–neprilysin inhibitors (ARNIs), and *β*-blockers was significantly lower in the SMuRF-less group than in the SMuRFs group (all *P* < 0.05), whereas the use of antiplatelet agents, nitroglycerin, and nicorandil was comparable between groups. Detailed medication data are shown in Table 2.

**Table 2:** In-hospital pharmacological therapy.

Variables	SMuRF-less	SMuRFs	Total	*P*
	(221, 21.5%)	(806, 78.4%)	(1,027, 100%)	
Aspirin	215 (97.2%)	794 (98.5%)	1,009 (98.2%)	0.347
P2Y12 inhibitor	216 (97.7%)	798 (99.0%)	1,014 (98.7%)	0.248
Statins	211 (95.4%)	800 (99.2%)	1,011 (98.4%)	<0.001
LMWH	131 (59.2%)	618 (76.6%)	749 (72.9%)	<0.001
nitroglycerin	146 (66.0%)	534 (66.2%)	680 (66.2%)	0.958
Nicorandil	66 (29.8%)	239 (29.6%)	305 (29.6%)	0.951
ACEIs/ARBs/ARNIs	132 (59.7%)	552 (68.4%)	684 (66.6%)	0.014
*β*- blocker	171 (77.3%)	697 (86.4%)	868 (84.5%)	0.001

P2Y12 inhibitor, clopidogrel or ticagrelor; LMWH, Low Molecular Weight Heparin; ACEI, Angiotensin-Converting Enzyme Inhibitor; ARB, Angiotensin II Receptor Blocker; ARNI, Angiotensin Receptor Neprilysin Inhibitor.

### 3.2 In-hospital clinical outcomes and post-procedural caIMR

In-hospital clinical outcomes are summarized in Table 3. The overall incidence of in-hospital MACE was significantly higher in the SMuRF-less group than in the SMuRFs group (15.3% vs. 7.0%, *P* < 0.001). Among the individual components of MACE, the incidence of malignant arrhythmias was significantly higher in the SMuRF-less group (4.5% vs. 1.4%, *P* = 0.012).

**Table 3:** In-hospital clinical events.

Variables	SMuRF-less	SMuRFs	Total	*P*
	(221, 21.5%)	(806, 78.4%)	(1,027, 100%)	
In-hospital MACE	34 (15.3%)	57 (7.0%)	91 (8.8%)	<0.001
In-hospital death	5 (2.2%)	13 (1.6%)	18 (1.7%)	0.717
HF	12 (5.4%)	26 (3.2%)	38 (3.7%)	0.124
CS	11 (4.9%)	20 (2.4%)	31 (3.0%)	0.055
Ischemia-Driven Revascularization	1 (0.4%)	0 (0.0%)	1 (0.1%)	0.215
Cardiac Arrest	1 (0.4%)	7 (0.8%)	8 (0.7%)	0.507
Malignant Arrhythmia	10 (4.5%)	12 (1.4%)	22 (2.1%)	0.012

MACE, major adverse cardiovascular events; HF,Heart Failure; CS, Cardiogenic Shock.

Analysis of coronary angiography–derived index of microcirculatory resistance is presented in Table 4. No significant differences were observed between groups in post-procedural TIMI frame count or coronary angiography-derived fractional flow Reserve (caFFR). However, post-procedural caIMR was significantly higher in the SMuRF-less group compared with the SMuRFs group (35.42 ± 7.33 vs. 31.35 ± 7.38, *P* = 0.011). Accordingly, the incidence of CMVD was significantly higher in the SMuRF-less group than in the SMuRFs group (26.6% vs. 11.0%, *P* < 0.001).

**Table 4:** Coronary physiological assessment.

Variables	SMuRF-less	SMuRFs	Total	*P*
	(221, 21.5%)	(806, 78.4%)	(1,027, 100%)	
Post-procedural TIMI Frame Count	18.05 ± 8.35	17.64 ± 8.43	17.81 ± 8.30	0.455
Post-procedural caIMR	35.42 ± 7.33	31.35 ± 7.38	32.13 ± 7.47	0.011
Post-procedural caFFR	0.90 [0.86, 0.94]	0.90 [0.86, 0.94]	0.90 [0.86, 0.94]	0.587
CMVD	59 (26.6%)	89 (11.0%)	148 (14.4%)	<0.001

TIMI, Thrombolysis in Myocardial Infarction; caIMR,coronary angiography–derived index of microcirculatory resistance; caFFR, coronary angiography–derived fractional flow reserve; CMVD, coronary microvascular dysfunction; CMVD is defined as a post-procedural caIMR > 40.

### 3.3 Association between SMuRF-less status and post-procedural caIMR

Using post-procedural caIMR as the dependent variable, multivariable linear regression analysis demonstrated that SMuRF-less status was independently associated with higher post-procedural caIMR after adjustment for age, sex, symptom-to-door time, left anterior descending artery as the culprit vessel, and pre-procedural TIMI flow grade (B = 4.379, 95% CI: 3.192–5.565, *P* < 0.001). No significant multicollinearity was observed among the independent variables (all variance inflation factors <2). Detailed data are shown in Table 5.

**Table 5:** Linear regression between SMuRF-less status and post-procedural caIMR.

Variables	B	SE	β	95% CI	*P*	*VIF*
SMuRF-less status	4.379	0.605	0.240	3.193– 5.565	<0.001	1.062
age	0.009	0.021	0.015	−0.031–0.049	0.665	1.151
male	0.432	0.615	0.024	−0.775–1.640	0.482	1.105
STD time	0.000	0.005	−0.002	−0.010–0.009	0.951	1.027
Culprit vessel LAD	0.199	0.490	0.013	−0.764–1.162	0.685	1.033
Pre-procedural TIMI flow grade	−0.057	0.210	−0.009	−0.468–0.355	0.786	1.044

SMuRF-less, without standard modifiable cardiovascular risk factors; STD time, symptom-to-door time; LAD, Left Anterior Descending artery; TIMI, Thrombolysis in Myocardial Infarction; Adjusted confounders in this study were selected based on clinical relevance, while variables with potential mediating effects were not included in the adjustment.

### 3.4 Predictive power of post-procedural caIMR for in-hospital MACE

In multivariable logistic regression analysis, post-procedural caIMR remained independently associated with an increased risk of in-hospital MACE after adjustment for SMuRF status, age, sex, symptom-to-door time, left anterior descending artery as the culprit vessel, and pre-procedural TIMI flow grade (OR = 1.063, 95% CI: 1.025–1.101, *P* = 0.001). SMuRF-less status was also independently associated with in-hospital MACE (OR = 2.094, 95% CI: 1.211–3.619, *P* = 0.008). None of the remaining covariates showed a significant association with in-hospital MACE in the fully adjusted model. Detailed data are shown in Table 6.

**Table 6:** Logistic regression for post-procedural caIMR and in-hospital MACE.

Variables	OR	95% CI	*P*
SMuRF-less status	2.094	1.211–3.619	0.008
Post-procedural caIMR	1.063	1.025–1.101	0.001
Age	1.007	0.986–1.029	0.501
Male	1.349	0.707–2.575	0.363
STD time	0.997	0.989–1.005	0.509
Culprit vessel LAD	1.040	0.633–1.709	0.877
Pre-procedural TIMI flow grade 1	0.680	0.235–1.965	0.476
Pre-procedural TIMI flow grade 2	2.178	0.752–6.309	0.152
Pre-procedural TIMI flow grade 3	0.724	0.343–1.529	0.397

SMuRF-less, without standard modifiable cardiovascular risk factors; caIMR,coronary angiography–derived index of microcirculatory resistance; STD time, symptom-to-door time; LAD, Left Anterior Descending artery; TIMI, Thrombolysis in Myocardial Infarction; Adjusted confounders in this study were selected based on clinical relevance, while variables with potential mediating effects were not included in the adjustment.

### 3.5 Analysis of mediating effects of post-procedural caIMR

To evaluate whether post-procedural caIMR mediated the association between SMuRF-less status and in-hospital MACE, mediation analysis was performed using the PROCESS macro (Model 4). Bootstrap analyses demonstrated a statistically significant indirect effect of SMuRF-less status on in-hospital MACE through post-procedural caIMR across sequential models: Model 1 (unadjusted), *β*= 0.209 (95% bootstrap CI: 0.070–0.385); Model 2 (adjusted for age and sex), *β*= 0.196 (95% bootstrap CI: 0.058–0.362); and Model 3 (adjusted for age, sex, symptom-to-door time, left anterior descending artery as the culprit vessel, and pre-procedural TIMI flow grade), *β*= 0.284 (95% bootstrap CI: 0.101–0.498). These findings indicate that post-procedural caIMR partially mediates the association between SMuRF-less status and in-hospital MACE. Detailed data are shown in Table 7. Sensitivity analyses were performed by further adjusting for discharge medications, including statins, LMWH, ACEIs/ARBs/ARNIs, and *β*-blockers. The results remained largely consistent with the primary analyses (Supplementary Tables S3 and S4).

**Table 7:** Mediation analysis of post-procedural caIMR.

	Model 1			Model 2			Model 3		
Effect/Path	β	95%CI	*P*	β	95%CI	*P*	β	95% CI	*P*
Direct Effect	0.675	0.205 ∼ 1.146	0.005	0.559	0.075 ∼ 1.044	0.024	0.695	0.153 ∼ 1.237	0.012
Indirect Effect	0.209	0.070 ∼ 0.385	-	0.196	0.058 ∼ 0.362	-	0.284	0.101∼ 0.498	-
Path a: X → M	4.175	3.077 ∼ 5.273	<0.001	4.144	3.024 ∼ 5.263	<0.001	4.379	3.193∼5.565	<0.001
Path b: M → Y	0.049	0.019 ∼ 0.081	0.002	0.047	0.017 ∼ 0.078	0.003	0.065	0.029∼0.101	<0.001

Model 1, unadjusted; Model 2, adjusted for age, sex; Model 3, adjusted for age, sex, symptom-to-door time,Culprit vessel LAD, Pre-procedural TIMI flow grade; X, SMuRF-less status; M, post-procedural caIMR; Y, In-hospital major adverse cardiovascular events; Since the outcome variable is binary, PROCESS Model 4 did not provide an estimate of the total effect.The significance of the indirect effect was determined using a bias-corrected bootstrap 95% confidence interval that did not include zero. Because its sampling distribution typically does not follow a normal distribution, a traditional *P*-value is not reported.

## 4 Discussion

In the present study, we observed that STEMI patients without standard modifiable cardiovascular risk factors experienced significantly worse in-hospital outcomes despite the absence of traditional risk factors. Mediation analysis further demonstrated that elevated post-procedural caIMR partially mediated the association between SMuRF-less status and adverse in-hospital outcomes. This indirect effect remained statistically significant after adjustment for potential confounders (*β*= 0.284, 95% bootstrap CI: 0.101–0.498), suggesting that coronary microvascular dysfunction contributes, at least in part, to the unfavorable in-hospital prognosis observed in SMuRF-less STEMI patients. These findings highlight impaired microcirculatory function as a potential pathophysiological link underlying the so-called “prognostic paradox”in this population.

In recent years, multiple studies have consistently reported that SMuRF-less STEMI patients paradoxically exhibit worse short-term outcomes than their counterparts with traditional cardiovascular risk factors, thereby challenging conventional risk stratification paradigms centered on atherosclerotic risk burden (3, 20, 21). However, most prior investigations have largely focused on outcome descriptions, with limited exploration of the underlying mechanisms. From a pathophysiological perspective, although SMuRF-less STEMI patients lack established risk factors such as hypertension, diabetes mellitus, dyslipidemia, and smoking, the occurrence of acute myocardial infarction in this group may not be driven primarily by long-standing stable atherosclerotic burden. Instead, it may be more closely related to vulnerable plaque rupture, heightened thrombotic burden, exaggerated inflammatory responses, and vascular functional abnormalities (22). Intravascular imaging studies have suggested that SMuRF-less patients are more likely to harbor lipid-rich plaques with thin fibrous caps, which are prone to rupture and subsequent distal microembolization, leading to obstruction of the coronary microvasculature (23). It should be noted that SMuRF-less status does not indicate a complete absence of cardiovascular risk determinants, as other factors such as genetic predisposition, lifestyle characteristics, and metabolic abnormalities may also contribute to disease development. In addition, SMuRF-less individuals may be exposed to non-classical risk mechanisms that are not captured by traditional definitions, including sleep and psychological health disturbances, systemic inflammatory states, hypermetabolic conditions, prothrombotic tendencies, sex-specific factors, and genetic or epigenetic susceptibility (24). These factors exert their effects through distinct pathways to culminate in a common pathological outcome:CMVD, resulting in impaired myocardial perfusion despite successful restoration of epicardial coronary flow.Previous studies have suggested that patients with a SMuRF-less profile may be less likely to receive intensive primary or secondary preventive therapies in routine clinical practice (25–27), which may in turn be associated with reduced microvascular tolerance to ischemia–reperfusion injury and aggravated microvascular dysfunction during reperfusion.

The coronary angiography-derived index of microcirculatory resistance provides a quantitative assessment of microvascular resistance and has been shown to correlate well with invasively measured IMR, as well as with infarct size, myocardial recovery, and clinical outcomes (7, 8). In the present study, SMuRF-less STEMI patients exhibited significantly higher post-procedural caIMR values than patients with SMuRFs, indicating more severe microvascular dysfunction even after successful reperfusion of the culprit artery. This dissociation between epicardial vessel patency and effective tissue-level perfusion may represent a key mechanism contributing to the increased risk of in-hospital MACE observed in this population (28–30). Collectively, these findings suggest that elevated caIMR constitutes an important pathophysiological pathway linking SMuRF-less status to adverse in-hospital outcomes and underscore the limitations of relying solely on traditional risk factors for prognostic assessment.

In addition, SMuRF-less patients were less likely to receive guideline-recommended pharmacological therapies during hospitalization, including statins, anticoagulants, ACEIs/ARBs/ARNIs, and β-blockers. These treatment disparities may reflect clinical risk assessment and therapeutic decision-making influenced by the presence of traditional cardiovascular risk factors rather than representing conventional confounders (3, 31). Because most in-hospital medications are initiated after STEMI onset and emergency revascularization and may lie on the causal pathway between SMuRF status, microvascular dysfunction, and outcomes, they were not included in the primary mediation models to avoid overadjustment. Nevertheless, sensitivity analyses further adjusting for these medications yielded similar results, supporting the robustness of our findings.

Taken together, our findings suggest that SMuRF-less STEMI patients represent a clinically under-recognized high-risk subgroup, in whom adverse in-hospital outcomes may be partially attributable to pronounced microvascular dysfunction during the acute phase. Enhanced recognition of microcirculatory impairment in this population, together with refined early risk stratification and targeted therapeutic strategies, may help improve in-hospital outcomes beyond conventional risk factor-based approaches.

## 5 Limitations

Several limitations of this study should be acknowledged. First, this was a retrospective observational study. Although multiple clinically relevant confounders were carefully adjusted for in the regression and mediation models, the possibility of residual confounding from unmeasured variables cannot be completely excluded, which may have influenced the observed mediation pathway.Second, mediation analysis assumes a clear temporal sequence between the exposure, mediator, and outcome. In the present study, caIMR was derived from coronary angiography during the acute phase of STEMI and was temporally close to the occurrence of in-hospital adverse outcomes. Therefore, while the proposed mechanistic pathway is biologically plausible, causal interpretation remains limited and warrants further validation in prospective studies.Third, the relatively modest sample size and limited number of in-hospital events may have reduced the statistical power of the mediation analysis. Finally, caIMR is a continuous angiography-derived index reflecting overall coronary microvascular resistance but cannot differentiate specific microvascular pathological mechanisms, such as distal microembolization, endothelial dysfunction, or microvascular spasm (32–34). Further studies integrating multimodal assessments are needed to elucidate the underlying biological mechanisms.

## 6 Conclusion

In patients with STEMI undergoing PCI, those with a SMuRF-less profile experienced significantly worse in-hospital outcomes. Our findings indicate that elevated post-procedural caIMR, as a quantitative marker of CMVD, partially mediates the association between SMuRF-less status and adverse in-hospital events. These results highlight CMVD as an important pathophysiological contributor to the unfavorable early prognosis observed in SMuRF-less STEMI patients and suggest that assessment of microvascular function may provide incremental value for early risk stratification in this population.

## Data Availability

The datasets presented in this article are not readily available because the datasets generated and/or analyzed during the current study are not publicly available due to them containing information that could compromise the privacy of research participants. However, they are available from the corresponding author on reasonable request, subject to approval by the institutional ethics committee and the execution of a data use agreement. Requests to access the datasets should be directed to Yuanyuan Luo, luoyuanyuanxyfy@126.com.
