# Supplementary material for: Coronary microvascular dysfunction mediates in-hospital adverse outcomes in ST-segment elevation myocardial infarction patients without standard modifiable cardiovascular risk factors
Source: Front Cardiovasc Med. 2026 Apr 17;13:1808392. doi: 10.3389/fcvm.2026.1808392 (PMC13132752; doi:10.3389/fcvm.2026.1808392)
Supplement: Supplementary file 1 [file Datasheet1.docx]

**Supplementary Table S1 |** Laboratory findings at admission.

| **Variables** | **SMuRF-less** | **SMuRFs** | **Total** | ***P*** |
| --- | --- | --- | --- | --- |
|  | **（221，21.5%）** | **（806，78.4%）** | **（1027，100%）** |  |
| **WBC(×10^9^/L)** | 9.42±2.92 | 9.86±3.14 | 9.76±3.10 | 0.071 |
| **NEUT(×10^9^/L)** | 7.61±2.90 | 7.75±3.03 | 7.72±3.00 | 0.540 |
| **CRP(mg/L)** | 20.20±37.48 | 27.84±48.01 | 25.67±45.43 | 0.056 |
| **NT-pro-BNP(pg/mL)** | 992[385, 2282] | 1048[400, 2036] | 1042[398, 2093] | 0.874 |
| **hs-TnT(ng/L)** | 2522[896, 5121] | 2432[908, 4852] | 2488[907,5008] | 0.682 |
| **CK-MB(ng/mL)** | 81[11, 244] | 97[25, 238] | 95[22, 239] | 0.222 |
| **LDH(U/L)** | 602[406, 995] | 601[320, 950] | 601[339, 956] | 1.000 |
| **CREA(μmol/L)** | 61[54, 71] | 64[53, 77] | 64[53, 76] | 0.059 |
| **eGFR(mL/min)** | 112.4[95.9, 120] | 114.9[93, 120] | 114.3[93.4, 120] | 0.331 |
| **UA(μmol/L)** | 292.70±100.54 | 311.17±90.64 | 307.01±93.23 | 0.012 |
| **FIB(g/L)** | 3.14±1.17 | 3.26±1.28 | 3.23±1.25 | 0.242 |
| **DD(μg/mL)** | 0.34[0.17, 0.60] | 0.29[0.16, 0.60] | 0.30[0.17, 0.60] | 0.203 |
| **FBG(mmol/L)** | 5.94±1.37 | 6.92±2.94 | 6.52±2.47 | 0.001 |
| **HbA1c(%)** | 5.83±0.83 | 6.49±1.40 | 6.30±1.26 | ＜0.001 |
| **TC(mmol/L)** | 3.93±0.76 | 4.60±1.07 | 4.45±1.05 | ＜0.001 |
| **TG(mmol/L)** | 1.05±0.35 | 1.64±1.43 | 1.51±1.30 | ＜0.001 |
| **HDL-C(mmol/L)** | 1.11±0.40 | 1.13±0.48 | 1.13±0.47 | 0.531 |
| **LDL-C(mmol/L)** | 2.40±0.68 | 2.82±0.99 | 2.73±0.95 | ＜0.001 |
| **Lp(a)(mg/L)** | 301.44±241.43 | 271.76±213.10 | 278.04±219.60 | 0.093 |
| **LVEF(%)** | 51.5±6.9 | 51.4±8.4 | 51.4±8.0 | 0.975 |

WBC, white blood cell count; NEUT, neutrophil count; CRP, C-reactive protein; NT-pro-BNP, N-terminal pro-B-type natriuretic peptide; hs-TnT, high-sensitivity cardiac troponin T; CK-MB, creatine kinase-myocardial band; LDH, lactate dehydrogenase; CREA, creatinine; eGFR, estimated glomerular filtration rate; UA, uric acid; FIB, fibrinogen; DD, D-dimer; FBG, fasting blood glucose; HbA1c, glycated hemoglobin; TC, total cholesterol; TG, triglycerides; HDL-C, high-density lipoprotein cholesterol; LDL-C, low-density lipoprotein cholesterol; Lp(a), lipoprotein(a); LVEF, left ventricular ejection fraction assessed by echocardiography.

**Supplementary Table S2 |** Angiographic characteristics.

| **Variables** | **SMuRF-less** | **SMuRFs** | **Total** | ***P*** |
| --- | --- | --- | --- | --- |
|  | **（221，21.5%）** | **（806，78.4%）** | **（1027，100%）** |  |
| **Culprit vessel** |  |  |  |  |
| LAD | 123（55.6%） | 419（51.9%） | 542（52.7%） | 0.333 |
| LCX | 17（7.6%） | 118（14.6%） | 135（13.1%） | 0.007 |
| RCA | 71（32.1%） | 234（29.0%） | 305（29.6%） | 0.372 |
| **Degree of stenosis(%)** |  |  |  |  |
| LM | 0[0, 0] | 0[0, 0] | 0[0, 0] | 0.197 |
| LAD | 95[72.5, 100] | 95[75, 100] | 95[75, 100] | 0.676 |
| LCX | 50[20, 85] | 50[0,90] | 50[0,90] | 0.298 |
| RCA | 70[35, 100] | 70[30,99] | 70[30,99] | 0.143 |
| **Gensini score** | 32[16, 32] | 16[16, 40] | 32[16, 40] | 0.553 |
| **Multivessel disease** | | | | |
| 1 | 111（50.2%） | 413（51.2%） | 524（51.0%） | 0.789 |
| 2 | 71（32.1%） | 238（29.5%） | 309（30.0%） | 0.456 |
| 3 | 31（14.0%） | 137（17.2%） | 170（16.5%） | 0.290 |
| **Degree of culprit vessel stenosis(%)** |  |  |  |  |
| ＜50% | 1（0.5%） | 4（0.5%） | 5（0.5%） | 0.999 |
| 50%-69% | 1（0.5%） | 6（0.7%） | 7（0.7%） | 0.995 |
| 70%-89% | 14（6.3%） | 36（4.5%） | 50（4.9%） | 0.253 |
| 90%-98% | 35（15.8%） | 154（19.1%） | 189（18.4%） | 0.266 |
| 99%-100% | 170（76.9%） | 606（75.2%） | 776（75.6%） | 0.595 |
| **Procedural time（H）** | 1.11±0.45 | 1.13±0.43 | 1.12±0.44 | 0.764 |
| **Pre-procedural TIMI flow grade** | 1.56±0.58 | 1.46±1.02 | 1.52±0.83 | 0.331 |
| 0 | 156（70.5%） | 480（59.5%） | 636（61.6%） | 0.003 |
| 1 | 14（6.3%） | 65（8.0%） | 79（7.6%） | 0.393 |
| 2 | 3（1.3%） | 30（3.7%） | 33（3.2%） | 0.077 |
| 3 | 21（9.5%） | 144（17.8%） | 165（16.0%） | 0.003 |
|  |  |  |  |  |
|  |  |  |  |  |

LAD, Left Anterior Descending artery; LCX, Left Circumflex artery; RCA, Right Coronary Artery; LM, Left Main artery; TIMI, Thrombolysis in Myocardial Infarction.

**Supplementary Table S3 |** Sensitivity analysis of logistic regression for post-procedural caIMR and in-hospital MACE.

| **Variables** | **OR** | **95% CI** | ***P*** |
| --- | --- | --- | --- |
| **SMuRF-less status** | 2.237 | 1.266–3.951 | 0.006 |
| **Post-procedural caIMR** | 1.062 | 1.024–1.100 | 0.001 |
| **Age** | 1.007 | 0.986–1.029 | 0.492 |
| **Male** | 1.313 | 0.688–2.507 | 0.409 |
| **STD time** | 0.998 | 0.990–1.006 | 0.553 |
| **Culprit vessel LAD** | 1.044 | 0.634–1.720 | 0.865 |
| **Pre-procedural TIMI flow grade 1** | 0.646 | 0.222–1.880 | 0.423 |
| **Pre-procedural TIMI flow grade 2** | 2.133 | 0.731–6.223 | 0.165 |
| **Pre-procedural TIMI flow grade 3** | 0.721 | 0.340–1.529 | 0.393 |
| **Statins** | 2.384 | 0.289–19.637 | 0.419 |
| **LMWH** | 1.435 | 0.789–2.609 | 0.236 |
| **ACEIs/ARBs/ARNIs** | 0.774 | 0.463–1.293 | 0.328 |
| **β- blocker** | 0.965 | 0.492–1.895 | 0.918 |

SMuRF-less, without standard modifiable cardiovascular risk factors; caIMR, coronary angiography–derived index of microcirculatory resistance; STD time, symptom-to-door time; LAD, Left Anterior Descending artery; TIMI, Thrombolysis in Myocardial Infarction; LMWH, Low Molecular Weight Heparin; ACEI, Angiotensin-Converting Enzyme Inhibitor; ARB, Angiotensin II Receptor Blocker; ARNI, Angiotensin Receptor Neprilysin Inhibitor; Adjusted confounders in this study were selected based on clinical relevance, while variables with potential mediating effects were not included in the adjustment.

**Supplementary Table S4 |** Sensitivity analysis of mediation analysis of post-procedural caIMR.

| **Effect/Path** | **β** | **95%CI** | **P** |
| --- | --- | --- | --- |
| **Direct Effect** | 0.752 | 0.188-1.316 | 0.009 |
| **Indirect Effect** | 0.289 | 0.106- 0.521 | - |
| **Path a: X → M** | 4.513 | 3.289-5.736 | <0.001 |
| **Path b: M→ Y** | 0.064 | 0.028-0.100 | 0.001 |

Adjusted for age, sex, symptom-to-door time, culprit vessel (LAD), pre-procedural TIMI flow grade, and the use of statins, LMWH, ACEIs/ARBs/ARNIs, and β-blockers; X, SMuRF-less status; M, post-procedural caIMR; Y, In-hospital major adverse cardiovascular events; Since the outcome variable is binary, PROCESS Model 4 did not provide an estimate of the total effect. The significance of the indirect effect was determined using a bias-corrected bootstrap 95% confidence interval that did not include zero. Because its sampling distribution typically does not follow a normal distribution, a traditional P-value is not reported.
